# Epidemiology of Masked and White-Coat Hypertension: The Family-Based SKIPOGH Study

**DOI:** 10.1371/journal.pone.0092522

**Published:** 2014-03-24

**Authors:** Heba Alwan, Menno Pruijm, Belen Ponte, Daniel Ackermann, Idris Guessous, Georg Ehret, Jan A. Staessen, Kei Asayama, Philippe Vuistiner, Sandrine Estoppey Younes, Fred Paccaud, Grégoire Wuerzner, Antoinette Pechere-Bertschi, Markus Mohaupt, Bruno Vogt, Pierre-Yves Martin, Michel Burnier, Murielle Bochud

**Affiliations:** 1 Institute of Social and Preventive Medicine (IUMSP), University Hospital of Lausanne, Lausanne, Switzerland; 2 Service of Nephrology, University Hospital of Lausanne, Lausanne, Switzerland; 3 Service of Nephrology, Department of Specialties, University Hospital of Geneva, Geneva, Switzerland; 4 Clinic for Nephrology, Hypertension and Clinical Pharmacology, Inselspital, Bern University Hospital and University of Bern, Bern, Switzerland; 5 Unit of Population Epidemiology, University Hospital of Geneva, Geneva, Switzerland; 6 Department of Cardiology, University Hospital of Geneva, Geneva, Switzerland; 7 Studies Coordinating Centre, Division of Hypertension and Cardiovascular Rehabilitation, Department of Cardiovascular Diseases, University of Leuven, Leuven, Belgium; 8 Department of Epidemiology, Maastricht University, Maastricht, The Netherlands; 9 Department of Planning for Drug Development and Clinical Evaluation, Tohoku University Graduate School of Pharmaceutical Sciences, Sendai, Japan; 10 Department of Community Medicine and Primary Care and Emergency Medicine, University Hospital of Geneva, Geneva, Switzerland; University Heart Center, Germany

## Abstract

**Objective:**

We investigated factors associated with masked and white-coat hypertension in a Swiss population-based sample.

**Methods:**

The Swiss Kidney Project on Genes in Hypertension is a family-based cross-sectional study. Office and 24-hour ambulatory blood pressure were measured using validated devices. Masked hypertension was defined as office blood pressure<140/90 mmHg and daytime ambulatory blood pressure≥135/85 mmHg. White-coat hypertension was defined as office blood pressure≥140/90 mmHg and daytime ambulatory blood pressure<135/85 mmHg. Mixed-effect logistic regression was used to examine the relationship of masked and white-coat hypertension with associated factors, while taking familial correlations into account. High-normal office blood pressure was defined as systolic/diastolic blood pressure within the 130–139/85–89 mmHg range.

**Results:**

Among the 652 participants included in this analysis, 51% were female. Mean age (±SD) was 48 (±18) years. The proportion of participants with masked and white coat hypertension was respectively 15.8% and 2.6%. Masked hypertension was associated with age (odds ratio (OR) = 1.02, p = 0.012), high-normal office blood pressure (OR = 6.68, p<0.001), and obesity (OR = 3.63, p = 0.001). White-coat hypertension was significantly associated with age (OR = 1.07, p<0.001) but not with education, family history of hypertension, or physical activity.

**Conclusions:**

Our findings suggest that physicians should consider ambulatory blood pressure monitoring for older individuals with high-normal office blood pressure and/or who are obese.

## Introduction

In the last few decades, the development of techniques that enable measurement of blood pressure (BP) outside the physician's office using either ambulatory or home BP has allowed individuals to be classified into four BP categories: sustained normotension (individuals who have normal office and out-of-office BP), sustained hypertension (individuals who have hypertension by both methods), white-coat hypertension (individuals who have office hypertension but normal out-of-office BP), and masked hypertension (individuals who have out-of-office hypertension but normal office BP) [1].

The term masked hypertension (MH) was first coined by Pickering et al in 2002 [2], [3]. The prevalence of MH may vary from 8 to 48% depending on the study methods and population [4]. Longitudinal studies have demonstrated that individuals with MH have higher cardiovascular disease (CVD) morbidity than normotensive persons, independent of other CVD risk factors [1], [5], [6]. Moreover, persons with MH have higher BP variability than normotensive individuals [7], and tend to subsequently develop sustained hypertension (SH) [8]. Identifying risk factors for MH may allow clinicians to select patients who should undergo ambulatory BP monitoring (ABPM) [4]. It has been previously shown that male sex [6], [9], age [10], [11], body mass index (BMI) [12], smoking and alcohol intake [11], high-normal office BP [11], and a low educational level [9] are associated with MH in the general population.

On the other hand, the clinical significance of white-coat hypertension (WCH) remains debated to this day [13]. Although the vast majority of studies have shown that individuals with WCH tend to have the same CVD morbidity as normotensive persons [14], [15], [16], some studies have demonstrated that individuals with WCH can have a CVD event rate comparable to individuals with sustained hypertension (SH) [17], [18], or are at a higher risk of developing SH with time [19]. If WCH is a truly benign condition, unnecessarily treating persons with WCH will have both clinical and financial consequences [13]. Factors that have been shown to be associated with WCH are age, hypertension of recent onset, and low education [13], [20].

Increasing research has been dedicated to better understanding the factors associated with MH. However, only a small number of studies using 24-hour ABPM have been conducted on a population-based level [6], [9], [12], [16], [21], [22], [23], with some being performed among individuals with a limited age range. We explored factors associated with masked hypertension and white-coat hypertension within the multicultural setting of Switzerland. We aimed to provide clinically useful indicators for these conditions in a population- and family-based multicentric sample of Swiss adults aged 18 years and older.

## Methods

### Ethics statement

All study participants provided written informed consent. The SKIPOGH study was approved by the Human Research Ethics Committee, Lausanne University Hospital and University of Lausanne (Lausanne, Switzerland), by the Ethics Committee for the Research on Human Beings, Geneva University Hospitals (Geneva, Switzerland), and by the Ethics Committee of the Canton of Bern, (Bern, Switzerland).

### Study population and design

SKIPOGH (Swiss Kidney Project on Genes in Hypertension) is a family and population-based cross-sectional study that examines the genetic determinants of BP. SKIPOGH is part of the larger family-based international EPOGH study (European Project on Genes in Hypertension). SKIPOGH employs the same methodology as that implemented and validated in the EPOGH study [24].

SKIPOGH is a multi-centre study with participants being recruited in the cantons of Bern and Geneva, and the city of Lausanne. Detailed methods have been previously described [25], [26]. Briefly, recruitment began in December 2009 and ended in April 2012 in Lausanne, in October 2012 in Geneva, and in April 2013 in Bern. Different strategies were used to draw up random samples of the population in each study centre. Index cases were randomly selected from the population-based CoLaus study [27] in Lausanne, and from the population-based Bus Santé study in Geneva [28]. In Bern, index participants were randomly selected using the cantonal phone directory. Inclusion criteria were as follows: (a) minimum age of 18 years; (b) of European descent (defined as having both parents and grandparents born in a restricted list of countries); (c) at least one, and ideally three, first degree family members also willing to participate in the study. Participation rate was 20% in Lausanne, 22% in Geneva, and 21% in Bern.

Participants filled in a standardized questionnaire at home. The questionnaire focused on a variety of issues including lifestyle habits as well as medical and drug history. The study visit was performed in the morning after an overnight fast. Electrolytes, kidney-function test, and blood glucose were measured in local laboratories using standard clinical laboratory methods. Participants were also asked to collect a 24-hour urine sample for the measurement of urinary volume, electrolytes, and the excretion of albumin, urea and creatinine. The CKD-EPI formula was used to calculate the estimated glomerular filtration rate (eGFR) [29]. Body weight and height were measured using precision electronic scales (Seca, Hamburg, Germany).

### Blood pressure measurements

BP was measured with a non-mercury auscultatory sphygmomanometer (A&D UM-101, A&D Company, Ltd., Toshima Ku, Tokyo, Japan) that has passed the International Protocol for validation of BP measuring devices of the European Society of Hypertension (ESH) [30]. This device has also been internally validated by our research group [31]. BP was measured after 10 minutes of rest in the sitting position in each arm. Subsequently, five consecutive BP measurements were taken on the side with the highest BP [13]. In this paper, each subject's office BP was defined as the mean of the last four office BP readings. 24-hour APBM was measured using a validated Diasys Integra device (Novacor, Rueil-Malmaison, France) that has fulfilled the validation criteria set forth by the British Hypertension Society and Association for the Advancement of Medical Instrumentation (AAMI) protocols [32]. Measurements were taken every 15 minutes during the day, and every 30 minutes during the night (from 10 pm to 7 am). Participants were included in the analyses if they had at least fourteen systolic BP (SBP) and diastolic BP (DBP) measurements during the day and at least seven readings during the night, in accordance with ESH recommendations [13]. 75 participants were excluded due to insufficient 24-hour BP readings. Day and night periods were obtained from participants' diaries over the 24-hour period. BP outliers were identified according to the editing criteria adopted by A&D software [33] and subsequently excluded from the analyses. Outliers were defined as SBP >280 mmHg, SBP <60 mmHg, DBP >200 mmHg, DBP <40 mmHg, heart rate >200 beats/min, heart rate <40 beats/min, or DBP ≥ SBP. If one of the BP measures was categorized as an outlier (e.g. SBP), then the other measures taken at the same instant in time (e.g. DBP and heart rate) were also excluded. On average, 3 BP readings were categorized as outliers per participant over the 24-hour period. Mean BP readings were subsequently calculated using the valid 24-hour, daytime, and night-time measurements.

Sustained normotension (NT) was defined as office BP <140/90 and daytime ambulatory BP <135/85 mmHg. MH was defined as office BP <140/90 mmHg and daytime ambulatory BP ≥135/85 mmHg. WCH was defined as office BP ≥140/90 mmHg and daytime ambulatory BP <135/85 mmHg [4], [13]. SH was defined as office BP ≥140/90 mmHg and daytime ambulatory BP ≥135/85 mmHg, or if the participant reported taking an anti-hypertensive medication. Accordingly, individuals on anti-hypertensive medication (n = 105) were excluded from the NT, WCH, and MH categories. For certain sensitivity analyses, SH was re-defined as having both office and ambulatory hypertension while excluding individuals who were on anti-hypertensive medication.

### Associated factors

High-normal office BP was defined as an office SBP between 130-139 mmHg and office DBP between 85–89 mmHg [34]. High-normal office BP was only assessed among normotensive individuals and individuals with MH as, by definition, participants with WCH and SH already have office hypertension. Smoking was defined as responding yes to the question “Do you currently smoke?” Alcohol consumption was defined as consuming at least one alcoholic beverage per week. BMI was calculated as weight (kilogram) divided by height squared (meter). BMI categories were defined as follows: normal-weight (<25 kg/m^2^), overweight (25–30 kg/m^2^), and obesity (≥30 kg/m^2^). Physical activity was assessed as a continuous variable by asking participants to report the number of hours per week they spend playing sports. Albuminuria was defined as a urinary albumin excretion ≥20 μg/min [35]. Education was classified into three categories: up to primary education, secondary education, and higher education (e.g. university or equivalent). Participants were asked to report if one of their parents or siblings is known to have hypertension or has suffered from a myocardial infarction (MI) or stroke.

### Analyses

All statistical analyses were conducted using Stata 12.0 (Stata College Station, TX). Complete case analysis was conducted on participants from Lausanne and Geneva, and participants from Bern up until 2011. Differences in baseline characteristics across BP categories were tested using Fisher's exact test for categorical variables and a 3 degrees of freedom likelihood-ratio test from a linear regression model for continuous variables. The ambulatory and office means for SBP, DBP, and heart rate were adjusted for age, sex, BMI, and study centre using mixed linear models, and a likelihood-ratio test was used to test the differences across BP categories, while taking familial correlations into account. Mixed-effect logistic regression was used to examine the relationship of MH, WCH, and SH with associated factors, while also taking familial correlations into account. For the latter analyses, normotension was used as the reference category.

## Results

Complete case analysis of the data resulted in 652 participants with 51% of the sample population being female. Mean age was 48 (±18) years. 15.0% of individuals had office hypertension (i.e. had an office BP ≥140/90 mmHg) and 28.2% had daytime ambulatory hypertension (i.e. had a daytime ambulatory BP ≥135/85 mmHg). Table 1 displays the descriptive characteristics of the study population for each BP category. Individuals with WCH were, on average, twenty years older than normotensive individuals, but had similar age as individuals with SH. 40.8% of MH individuals had high-normal office BP as compared to 7.8% of normotensive individuals (p<0.001).

**Table 1:** Descriptive statistics of the study population (n = 652).

	Normotension	White-coat hypertension	Masked hypertension	Sustained hypertension	P^a^
Proportion	386 (59.2)	17 (2.6)	103 (15.8)	146 (22.4)	
Age (years)	41.9 (16.9)	61.3 (15.6)	48.7 (13.6)	62.0 (12.3)	<0.001
Sex (female)	216 (56.0)	7 (41.2)	43 (41.8)	64 (43.8)	0.012
BMI (kg/m^2^)	23.7 (3.8)	26.5 (3.9)	26.5 (4.4)	27.1 (4.0)	<0.001
eGFR (ml/min/1.73 m^2^)	100.6 (17.1)	87.2 (15.2)	93.9 (17.2)	87.5 (16.0)	<0.001
Smoking (yes)	95 (24.6)	1 (5.9)	20 (19.4)	29 (19.9)	0.199
Alcohol consumption (yes)	234 (60.6)	11 (64.7)	67 (65.1)	99 (67.8)	0.464
High-normal office BP	30 (7.8)		42 (40.8)		<0.001
Microalbuminuria ≥20 μg/min	17 (4.4)	2 (11.8)	9 (8.7)	12 (8.2)	0.089
Education (up to primary)	49 (12.7)	3 (17.7)	9 (8.7)	24 (16.4)	0.542
Secondary	196 (50.8)	8 (47.1)	53 (51.5)	77 (52.7)	
Higher education	141 (36.5)	6 (35.3)	41 (39.8)	45 (30.8)	
Physical activity (hours/week)	3.1 (4.4)	2.7 (3.0)	2.2 (2.7)	2.3 (2.9)	0.034
Family history of hypertension	182 (47.2)	11 (64.7)	48 (46.6)	91 (62.3)	0.008
Family history of MI or stroke	85 (22.0)	7 (41.2)	35 (34.0)	75 (51.4)	<0.001

Data are mean (standard deviation) or n (%).

a Comparison between the four blood pressure categories; BMI: body mass index, eGFR: estimated glomerular filtration rate, BP: blood pressure, MI: myocardial infarction.

**Figure 1:**
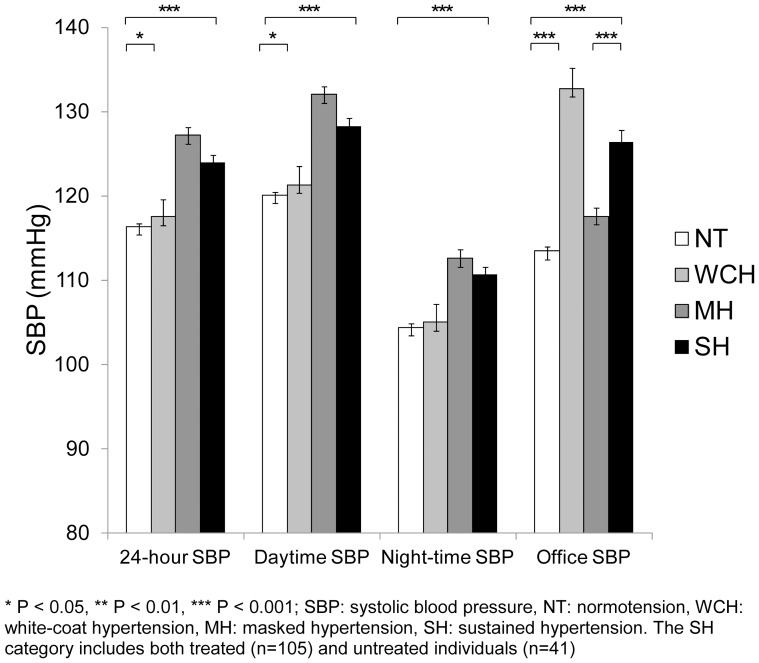
Mean (and standard error) ambulatory and office systolic blood pressure (SBP) by blood pressure category.

**Figure 2:**
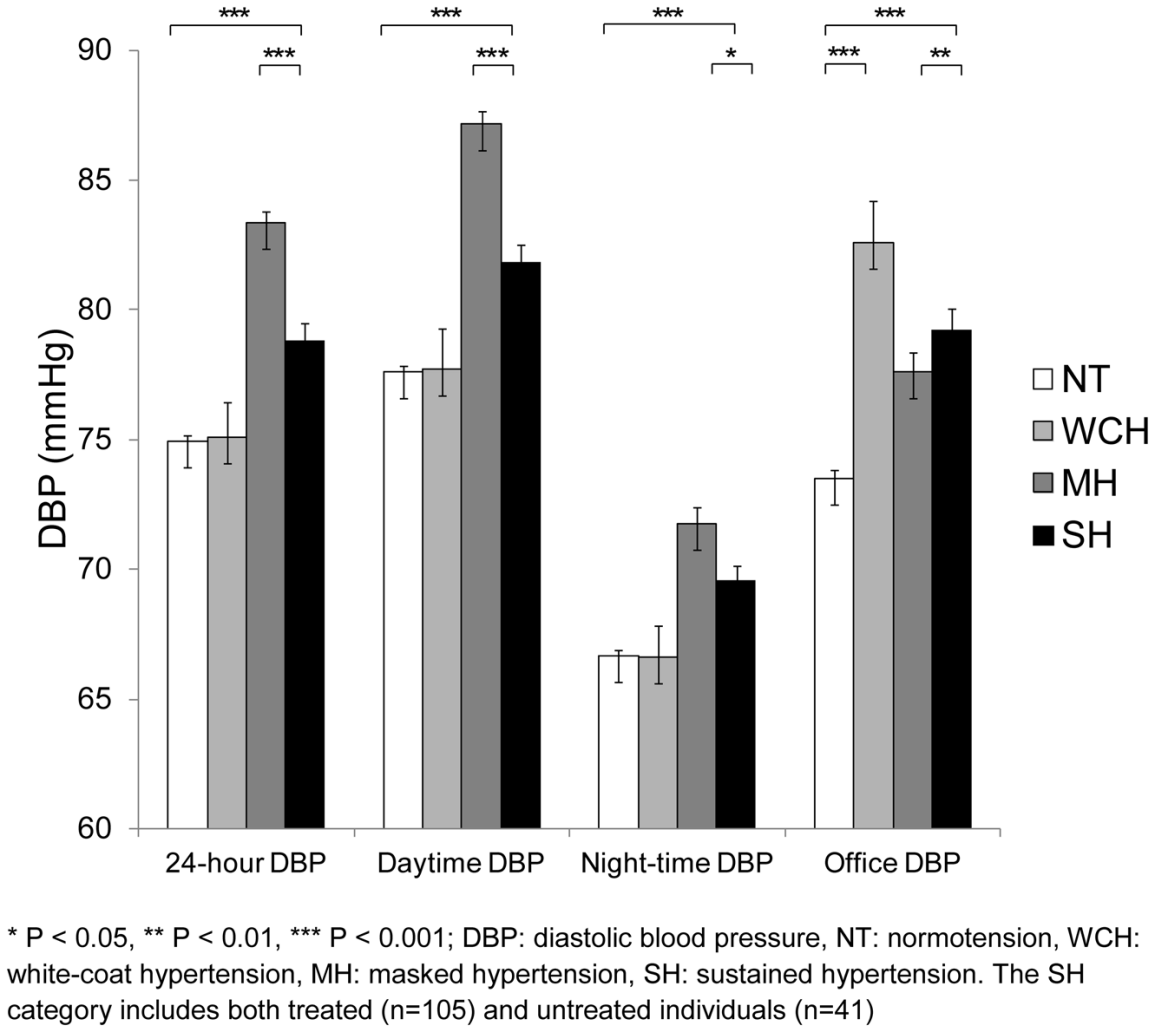
Mean (and standard error) ambulatory and office diastolic blood pressure (DBP) by blood pressure category.

**Figure 3:**
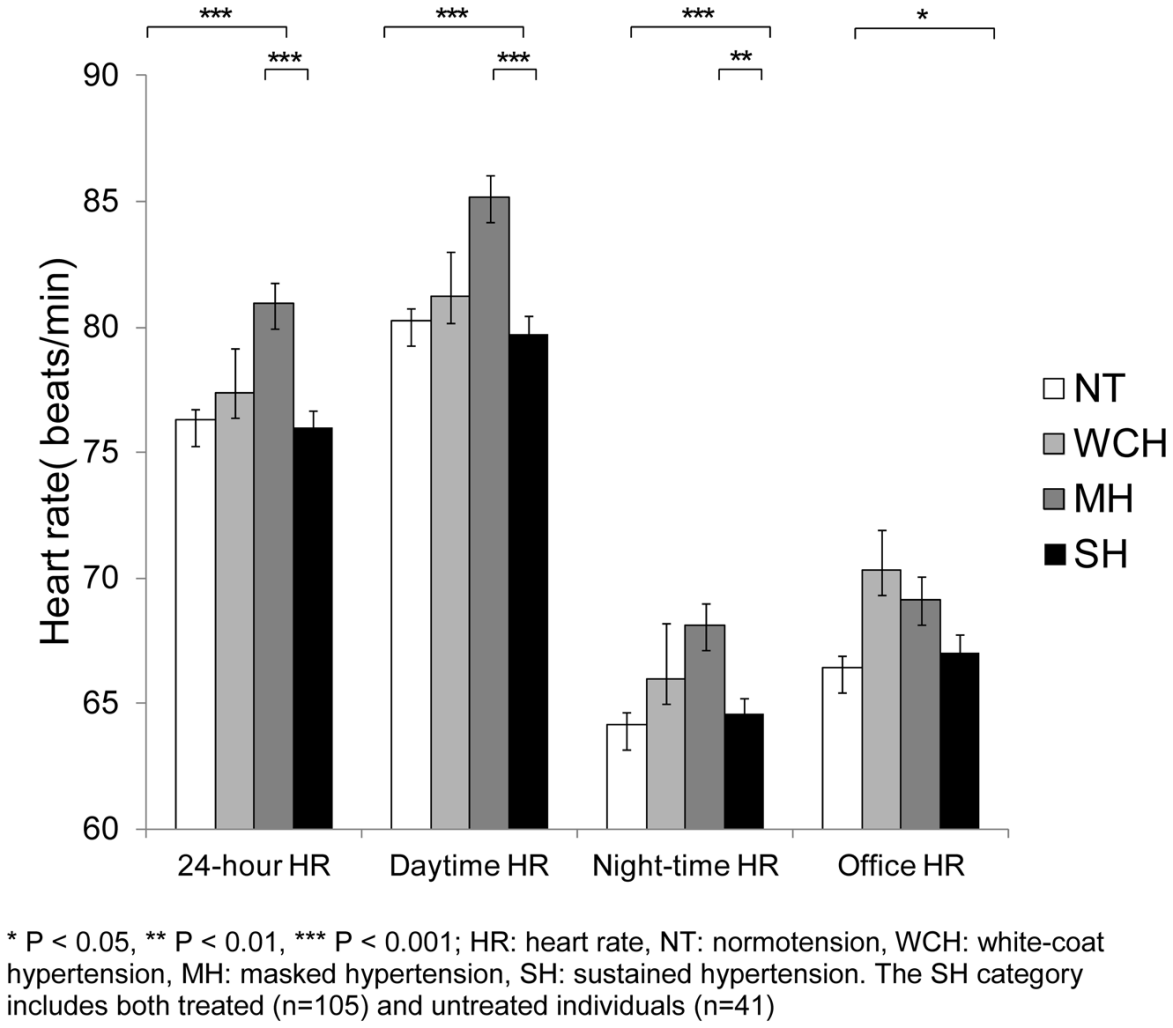
Mean (and standard error) ambulatory and office heart rate (HR) by blood pressure category.

Figures 1, 2, 3 display mean ambulatory and office SBP, DBP, and heart rate by BP category (exact values of means, standard errors and p values can be found in Table S1). Individuals with MH and SH tended to have the highest 24-hour BP values as compared to the other BP categories. As expected, individuals with WCH had the highest office BP levels (p<0.001). Individuals with MH had the highest 24-hour, daytime, and night-time heart rate (p<0.01).

Univariate and multivariable logistic regression analyses were performed to assess the associations between MH and selected factors, with normotensive individuals acting as the reference group (Table 2). In multivariable analysis, age and overweight/obesity were positively associated with MH (odds ratio (OR): 1.02, p = 0.012 for age; OR: 3.63, p = 0.001 for obesity). The odds of having MH were 6.7 times greater among individuals with high-normal office BP (p<0.001) as compared to individuals with an office BP <130/85 mmHg. Physical activity (hours per week) was inversely associated with MH (OR: 0.92; 95% confidence interval (CI): 0.85–0.99). There was no statistically significant association for smoking, alcohol consumption, or educational level. Moreover, we did not find a significant association between MH and fasting serum glucose, serum sodium and potassium, 24-hour urinary sodium and potassium excretion, microalbuminuria, and eGFR (results not shown).

**Table 2:** Factors associated with masked hypertension (n = 103).

		Univariate				Age, sex, centre, BMI-adjusted			
	n	OR	95% CI		P	OR	95% CI		P
Age (years)		1.03	1.01	1.04	<0.001	1.02	1.00	1.03	0.012
Sex (female)	43	0.56	0.36	0.88	0.011	0.63	0.39	1.02	0.058
High-normal office BP	42	8.17	4.75	14.04	<0.001	6.68	3.72	12.02	<0.001
Smoking (yes)	20	0.74	0.43	1.27	0.271	0.78	0.44	1.40	0.404
Alcohol consumption (yes)	67	1.21	0.77	1.90	0.412	1.01	0.61	1.66	0.981
Normal-weight	40								
Overweight	45	3.54	2.10	5.99	<0.001	2.99	1.74	5.15	<0.001
Obese	18	4.26	2.09	8.66	<0.001	3.63	1.73	7.60	0.001
Physical activity (hours/week)		0.93	0.86	0.99	0.035	0.92	0.85	0.99	0.023
Education (up to primary)	9								
Secondary	53	1.47	0.68	3.20	0.329	1.48	0.63	3.48	0.364
Higher education	41	1.58	0.71	3.53	0.261	2.02	0.83	4.91	0.121
Family history of hypertension	48	0.98	0.63	1.51	0.921	0.81	0.50	1.31	0.391
Family history of MI* or stroke	35	1.82	1.14	2.93	0.013	1.49	0.87	2.57	0.150

*MI: myocardial infarction. Analyses included people with masked hypertension and people with normotension as a comparison group. Total sample size for this analysis is 489 participants.

Table 3 displays the relationship between WCH and associated factors. As only one person reported smoking among individuals with WCH, smoking was not included as a covariate in these analyses. Similarly to MH, WCH was positively associated with age (p<0.001).

**Table 3:** Factors associated with white-coat hypertension (n = 17).

		Univariate				Age, sex, centre, BMI-adjusted			
Variable	n	OR	95% CI		P	OR	95% CI		P
Age (years)		1.07	1.04	1.11	<0.001	1.07	1.03	1.11	<0.001
Sex (female)	7	0.55	0.21	1.48	0.236	0.61	0.21	1.76	0.364
Alcohol consumption (yes)	11	1.19	0.43	3.29	0.736	0.95	0.31	2.95	0.935
Normal-weight	6								
Overweight	8	4.14	1.40	12.25	0.010	2.43	0.73	8.05	0.146
Obese	3	4.66	1.11	19.61	0.036	2.96	0.60	14.56	0.181
Physical activity (hours/week)		0.97	0.85	1.11	0.674	0.91	0.79	1.06	0.230
Education (up to primary)	3								
Secondary	8	0.67	0.17	2.61	0.560	0.85	0.19	3.89	0.834
Higher education	6	0.70	0.17	2.89	0.616	1.24	0.24	6.31	0.799
Family history of hypertension	11	2.05	0.75	5.67	0.164	2.15	0.65	7.15	0.213
Family history of MI* or stroke	7	2.48	0.92	6.71	0.074	1.32	0.35	4.94	0.678

*MI: myocardial infarction. Analyses included people with white-coat hypertension and people with normotension as a comparison group. Total sample size for this analysis is 403 participants.

The association between SH and selected factors is presented in Table 4. There was a positive association between SH and age, obesity, and a family history of hypertension, MI, or stroke (p<0.05). Physical activity was inversely associated with SH (OR: 0.91; 95% CI: 0.85–0.99).

**Table 4:** Factors associated with sustained hypertension (n = 146).

		Univariate				Age, sex, centre, BMI-adjusted			
Variable	n	OR	95% CI		P	OR	95% CI		P
Age (years)		1.09	1.07	1.11	<0.001	1.09	1.07	1.11	<0.001
Sex (female)	64	0.61	0.41	0.91	0.015	0.73	0.44	1.19	0.207
Smoking (yes)	29	0.72	0.44	1.19	0.196	1.33	0.72	2.47	0.360
Alcohol consumption (yes)	99	1.39	0.91	2.12	0.128	1.08	0.63	1.87	0.778
Normal-weight	43								
Overweight	75	5.58	3.48	8.94	<0.001	3.36	1.94	5.84	<0.001
Obese	28	6.26	3.30	11.90	<0.001	4.25	2.04	8.85	<0.001
Physical activity (hours/week)		0.93	0.88	0.99	0.028	0.91	0.85	0.99	0.022
Education (up to primary)	24								
Secondary	77	0.79	0.44	1.41	0.423	1.14	0.53	2.47	0.739
Higher education	45	0.65	0.35	1.20	0.171	1.67	0.71	3.91	0.240
Family history of hypertension	91	1.85	1.24	2.76	0.003	1.87	1.13	3.10	0.014
Family history of MI* or stroke	75	3.99	2.55	6.25	<0.001	2.09	1.24	3.53	0.006

*MI: myocardial infarction. Analyses included people with sustained hypertension and people with normotension as a comparison group. Total sample size for this analysis is 532 participants.

We also conducted sensitivity analyses to explore whether the factors associated with MH differed by study centre. Due to the limited number of individuals with MH in our study, we ran the analyses while grouping two study centres at a time (e.g. Geneva and Bern, Geneva and Lausanne, Lausanne and Bern). The factors associated with MH were very similar to our findings including all three centres (results not shown). We could not perform similar sensitivity analyses for WCH due to the limited number of participants identified as white-coat hypertensives.

## Discussion

Our study is the first to estimate the proportion of MH in a family- and population-based study in Switzerland. We found that one in six individuals in our sample had MH, a finding comparable to other population-based studies [12], [21], [22], [23]. Although the frequency of WCH in our study (3%) was lower than that normally reported in the literature, other population-based studies have also found a prevalence within the range of 3–5% [12], [36], [37]. This low WCH proportion may be attributable to the relatively young age of our study population, as WCH tends to occur more often in older individuals [38], as well as the clinical settings in which office BP was measured in this study (after ten minutes of rest). Moreover, mean office BP was calculated using the last four BP readings whilst disregarding the first BP measurement. Correspondingly, individuals with WCH were, on average, 20 years older than normotensive individuals in our study. Moreover, it has been shown that WCH is associated with a previous diagnosis of hypertension [38], and as the current study is population-based, only 16% of individuals reported being on anti-hypertensive medication. It should also be noted that the proportion of MH and WCH is directly related to the cut-offs used to define normal office and ambulatory BP [4]. Although most studies tend to use the thresholds of 140/90 mmHg for office BP and 135/85 mmHg for daytime ambulatory BP, some studies set their thresholds based on the population studied, use arbitrary thresholds, or employ thresholds that are identical for both office and ambulatory BP [4].

A growing body of evidence has found that ABPM is better correlated with target organ damage than office BP measurement [15], and that persons with MH have a CVD risk comparable to those with SH [14]. The odds of having MH was seven times greater among participants who had a high-normal office BP, a finding consistent with previous reports [3], [10], [11], [39]. Moreover, the proportion of MH among individuals with high-normal office BP was 31.8% as compared to 11.7% among individuals with an office BP of less than 130/85 mmHg. High-normal office BP may thus be of important clinical utility, as it is easy to measure and appears to be strongly associated with MH, thereby aiding physicians in selecting patients for ABPM. Shimbo et al hypothesized that although both ambulatory and office BP increase with age, ambulatory hypertension may precede office hypertension, whereas office BP rises but remains within the normal office BP range in the early stages [3]. In our study, 41% of individuals with MH had high-normal office BP. It is presumed that the office BP of these individuals will eventually increase leading to SH [3], suggesting that it would be advisable to screen these individuals for MH. Notably, just over half of the global burden of CVD that is attributable to BP occurs in individuals with high-normal BP [40]. Of note, in contrast to some earlier studies, MH in this paper was considered only among untreated individuals.

Some studies [11], [39], although not all [12], [16], [41], have found that smoking and alcohol consumption are correlated with MH and WCH. In our study, we did not find a significant association between smoking and alcohol consumption and BP status. In line with the existing literature [11], [22], [39], persons who were overweight or obese were more likely to have MH and SH. Moreover, physical activity was inversely associated with MH, consistent with a previous study that also found that MH was associated with a low physical activity score among nonsmokers [41].

We found that individuals with MH and SH had the highest ambulatory BP whereas individuals with WCH had the highest office BP. Interestingly, the mean ambulatory DBP of masked hypertensives was significantly higher than that of SH individuals. When treated individuals were excluded from the SH category, this was no longer the case. We also found that individuals with MH had the highest ambulatory heart rate. It has been postulated that MH is associated with a sympathetic overdrive, although the exact mechanisms underlying this sympathetic activation have to be further clarified [38], [42]. In our study, individuals with WCH had the highest office heart rate, which is compatible with previous results showing that individuals with WCH have an increased sympathetic activity [23], [43].

Our study has some limitations. The cross-sectional nature of this study limits inference on causality. There were relatively few participants who were classified as having WCH, limiting the statistical analyses that may be performed on this sub-group. Likewise, we were unable to stratify individuals in the SH category by treatment status due to the low number of participants who had SH and were not on anti-hypertensive medication. Participation rate was relatively low, raising the issue of selection bias and thus limiting the external validity of our results. However, there was no difference in terms of age and sex distribution between the participants to this study and those of the population-based sample from which they were drawn [25]. Moreover, each study centre used a different strategy to select study participants, which implies that selection biases, if any, are likely to differ across centers. Our sensitivity analyses demonstrated that results were similar when restricting the analyses to two centres instead of three centres. Furthermore, the factors we found to be associated with MH were similar to those previously published, which suggests that our findings have reasonable external validity. Strengths of this study include its population-and family-based nature and the relatively large sample size.

## Conclusion

We found that individuals with MH tend to share similar characteristics as patients with SH. Although there is currently a lack of randomized controlled trials that have investigated the benefits of treating patients with MH, it is plausible that by identifying individuals with MH, cardiovascular complications and target organ damage may potentially be avoided if management of hypertension is based on ambulatory BP [16]. Our results suggest that clinicians should suspect MH in an older (and/or overweight) patient with high-normal office BP.

## Supporting Information

Table S1 Mean ambulatory and office systolic blood pressure (SBP), diastolic blood pressure (DBP), and heart rate (HR) by blood pressure category.
(DOCX)

## References

[1] Angeli F, Reboldi G, Verdecchia P (2010) Masked hypertension: evaluation, prognosis, and treatment. Am J Hypertens 23: 941–948. 20508623 10.1038/ajh.2010.112

[2] Pickering TG, Davidson K, Gerin W, Schwartz JE (2002) Masked hypertension. Hypertension 40: 795–796. 12468559 10.1161/01.hyp.0000038733.08436.98

[3] Shimbo D, Newman JD, Schwartz JE (2012) Masked hypertension and prehypertension: diagnostic overlap and interrelationships with left ventricular mass: the Masked Hypertension Study. Am J Hypertens 25: 664–671. 22378035 10.1038/ajh.2012.15 PMC3668422

[4] Bobrie G, Clerson P, Menard J, Postel-Vinay N, Chatellier G, et al (2008) Masked hypertension: a systematic review. J Hypertens 26: 1715–1725. 18698202 10.1097/HJH.0b013e3282fbcedf

[5] Bjorklund K, Lind L, Zethelius B, Andren B, Lithell H (2003) Isolated ambulatory hypertension predicts cardiovascular morbidity in elderly men. Circulation 107: 1297–1302. 12628951 10.1161/01.cir.0000054622.45012.12

[6] Hansen TW, Jeppesen J, Rasmussen S, Ibsen H, Torp-Pedersen C (2006) Ambulatory blood pressure monitoring and risk of cardiovascular disease: a population based study. Am J Hypertens 19: 243–250. 16500508 10.1016/j.amjhyper.2005.09.018

[7] Cacciolati C, Tzourio C, Hanon O (2013) Blood pressure variability in elderly persons with white-coat and masked hypertension compared to those with normotension and sustained hypertension. Am J Hypertens 26: 367–372. 23382487 10.1093/ajh/hps054

[8] Mancia G, Bombelli M, Facchetti R, Madotto F, Quarti-Trevano F, et al (2009) Long-term risk of sustained hypertension in white-coat or masked hypertension. Hypertension 54: 226–232. 19564548 10.1161/HYPERTENSIONAHA.109.129882

[9] Schoenthaler AM, Schwartz J, Cassells A, Tobin JN, Brondolo E (2010) Daily interpersonal conflict predicts masked hypertension in an urban sample. Am J Hypertens 23: 1082–1088. 20616788 10.1038/ajh.2010.141

[10] Cacciolati C, Hanon O, Alperovitch A, Dufouil C, Tzourio C (2011) Masked hypertension in the elderly: cross-sectional analysis of a population-based sample. Am J Hypertens 24: 674–680. 21415840 10.1038/ajh.2011.23

[11] Hanninen MR, Niiranen TJ, Puukka PJ, Mattila AK, Jula AM (2011) Determinants of masked hypertension in the general population: the Finn-Home study. J Hypertens 29: 1880–1888. 21841499 10.1097/HJH.0b013e32834a98ba

[12] Hanninen MR, Niiranen TJ, Puukka PJ, Jula AM (2010) Comparison of home and ambulatory blood pressure measurement in the diagnosis of masked hypertension. J Hypertens 28: 709–714. 20061982 10.1097/HJH.0b013e3283369faa

[13] O'Brien E, Asmar R, Beilin L, Imai Y, Mallion JM, et al (2003) European Society of Hypertension recommendations for conventional, ambulatory and home blood pressure measurement. J Hypertens 21: 821–848. 12714851 10.1097/00004872-200305000-00001

[14] Fagard RH, Cornelissen VA (2007) Incidence of cardiovascular events in white-coat, masked and sustained hypertension versus true normotension: a meta-analysis. J Hypertens 25: 2193–2198. 17921809 10.1097/HJH.0b013e3282ef6185

[15] Bobrie G, Genes N, Vaur L, Clerson P, Vaisse B, et al (2001) Is “isolated home” hypertension as opposed to “isolated office” hypertension a sign of greater cardiovascular risk? Arch Intern Med 161: 2205–2211. 11575977 10.1001/archinte.161.18.2205

[16] Ohkubo T, Kikuya M, Metoki H, Asayama K, Obara T, et al (2005) Prognosis of “masked” hypertension and “white-coat” hypertension detected by 24-h ambulatory blood pressure monitoring 10-year follow-up from the Ohasama study. J Am Coll Cardiol 46: 508–515. 16053966 10.1016/j.jacc.2005.03.070

[17] Gustavsen PH, Hoegholm A, Bang LE, Kristensen KS (2003) White coat hypertension is a cardiovascular risk factor: a 10-year follow-up study. J Hum Hypertens 17: 811–817. 14704724 10.1038/sj.jhh.1001643

[18] Verdecchia P, Reboldi GP, Angeli F, Schillaci G, Schwartz JE, et al (2005) Short- and long-term incidence of stroke in white-coat hypertension. Hypertension 45: 203–208. 15596572 10.1161/01.HYP.0000151623.49780.89

[19] Bidlingmeyer I, Burnier M, Bidlingmeyer M, Waeber B, Brunner HR (1996) Isolated office hypertension: a prehypertensive state? J Hypertens 14: 327–332. 8723986 10.1097/00004872-199603000-00009

[20] Mancia G, Bombelli M, Seravalle G, Grassi G (2011) Diagnosis and management of patients with white-coat and masked hypertension. Nat Rev Cardiol 8: 686–693. 21826071 10.1038/nrcardio.2011.115

[21] Sega R, Trocino G, Lanzarotti A, Carugo S, Cesana G, et al (2001) Alterations of cardiac structure in patients with isolated office, ambulatory, or home hypertension: Data from the general population (Pressione Arteriose Monitorate E Loro Associazioni [PAMELA] Study). Circulation 104: 1385–1392. 11560854 10.1161/hc3701.096100

[22] Wang GL, Li Y, Staessen JA, Lu L, Wang JG (2007) Anthropometric and lifestyle factors associated with white-coat, masked and sustained hypertension in a Chinese population. J Hypertens 25: 2398–2405. 17984660 10.1097/HJH.0b013e3282efeee7

[23] Fagard RH, Stolarz K, Kuznetsova T, Seidlerova J, Tikhonoff V, et al (2007) Sympathetic activity, assessed by power spectral analysis of heart rate variability, in white-coat, masked and sustained hypertension versus true normotension. J Hypertens 25: 2280–2285. 17921823 10.1097/HJH.0b013e3282efc1fe

[24] Kuznetsova T, Staessen JA, Kawecka-Jaszcz K, Babeanu S, Casiglia E, et al (2002) Quality control of the blood pressure phenotype in the European Project on Genes in Hypertension. Blood Press Monit 7: 215–224. 12198337 10.1097/00126097-200208000-00003

[25] Ponte B, Pruijm M, Ackermann D, Vuistiner P, Eisenberger U, et al.. (2013) Reference Values and Factors Associated With Renal Resistive Index in a Family-Based Population Study. Hypertension. 10.1161/HYPERTENSIONAHA.113.02321 24126174

[26] Pruijm M, Ponte B, Ackermann D, Vuistiner P, Paccaud F, et al (2013) Heritability, determinants and reference values of renal length: a family-based population study. Eur Radiol 23: 2899–2905. 23712436 10.1007/s00330-013-2900-4

[27] Firmann M, Mayor V, Vidal PM, Bochud M, Pecoud A, et al (2008) The CoLaus study: a population-based study to investigate the epidemiology and genetic determinants of cardiovascular risk factors and metabolic syndrome. BMC Cardiovasc Disord 8: 6. 18366642 10.1186/1471-2261-8-6 PMC2311269

[28] Guessous I, Bochud M, Theler JM, Gaspoz JM, Pechere-Bertschi A (2012) 1999–2009 Trends in prevalence, unawareness, treatment and control of hypertension in Geneva, Switzerland. PLoS One 7: e39877. 22761919 10.1371/journal.pone.0039877 PMC3384604

[29] Levey AS, Stevens LA, Schmid CH, Zhang YL, Castro AF 3rd, et al (2009) A new equation to estimate glomerular filtration rate. Ann Intern Med 150: 604–612. 19414839 10.7326/0003-4819-150-9-200905050-00006 PMC2763564

[30] Stergiou GS, Giovas PP, Gkinos CP, Tzamouranis DG (2008) Validation of the A&D UM-101 professional hybrid device for office blood pressure measurement according to the International Protocol. Blood Press Monit 13: 37–42. 18199922 10.1097/MBP.0b013e3282c9acb0

[31] Pruijm MT, Wuerzner G, Glatz N, Alwan H, Ponte B, et al (2010) A new technique for simultaneous validation of two manual nonmercury auscultatory sphygmomanometers (A&D UM-101 and Accoson Greenlight 300) based on the International protocol. Blood Press Monit 15: 322–325. 20827175 10.1097/MBP.0b013e32833f56a8

[32] O'Brien E, Waeber B, Parati G, Staessen J, Myers MG (2001) Blood pressure measuring devices: recommendations of the European Society of Hypertension. BMJ 322: 531–536. 11230071 10.1136/bmj.322.7285.531 PMC1119736

[33] Winnicki M, Canali C, Mormino P, Palatini P (1997) Ambulatory blood pressure monitoring editing criteria: is standardization needed? Hypertension and Ambulatory Recording Venetia Study (HARVEST) Group, Italy. Am J Hypertens 10: 419–427. 9128208

[34] Mancia G, De Backer G, Dominiczak A, Cifkova R, Fagard R, et al (2007) 2007 Guidelines for the Management of Arterial Hypertension: The Task Force for the Management of Arterial Hypertension of the European Society of Hypertension (ESH) and of the European Society of Cardiology (ESC). J Hypertens 25: 1105–1187. 17563527 10.1097/HJH.0b013e3281fc975a

[35] Sarafidis PA, Riehle J, Bogojevic Z, Basta E, Chugh A, et al (2008) A comparative evaluation of various methods for microalbuminuria screening. Am J Nephrol 28: 324–329. 18046079 10.1159/000111825

[36] Sehestedt T, Jeppesen J, Hansen TW, Rasmussen S, Wachtell K, et al (2012) Can ambulatory blood pressure measurements substitute assessment of subclinical cardiovascular damage? J Hypertens 30: 513–521. 22241138 10.1097/HJH.0b013e32834f6f60

[37] Stergiou GS, Baibas NM, Kalogeropoulos PG (2007) Cardiovascular risk prediction based on home blood pressure measurement: the Didima study. J Hypertens 25: 1590–1596. 17620954 10.1097/HJH.0b013e3281ab6c69

[38] Trudel X, Brisson C, Larocque B, Milot A (2009) Masked hypertension: different blood pressure measurement methodology and risk factors in a working population. J Hypertens 27: 1560–1567. 19444141 10.1097/HJH.0b013e32832cb036

[39] Obara T, Ohkubo T, Funahashi J, Kikuya M, Asayama K, et al (2005) Isolated uncontrolled hypertension at home and in the office among treated hypertensive patients from the J-HOME study. J Hypertens 23: 1653–1660. 16093909 10.1097/01.hjh.0000178334.33352.56

[40] Lawes CM, Vander Hoorn S, Rodgers A (2008) International Society of H (2008) Global burden of blood-pressure-related disease, 2001. Lancet 371: 1513–1518. 18456100 10.1016/S0140-6736(08)60655-8

[41] Makris TK, Thomopoulos C, Papadopoulos DP, Bratsas A, Papazachou O, et al (2009) Association of passive smoking with masked hypertension in clinically normotensive nonsmokers. Am J Hypertens 22: 853–859. 19478792 10.1038/ajh.2009.92

[42] Grassi G, Seravalle G, Trevano FQ, Dell'oro R, Bolla G, et al (2007) Neurogenic abnormalities in masked hypertension. Hypertension 50: 537–542. 17620522 10.1161/HYPERTENSIONAHA.107.092528

[43] Bochud M, Bovet P, Vollenweider P, Maillard M, Paccaud F, et al (2009) Association between white-coat effect and blunted dipping of nocturnal blood pressure. Am J Hypertens 22: 1054–1061. 19629048 10.1038/ajh.2009.133

