# Supplementary material for: Epidemiology of Masked and White-Coat Hypertension: The Family-Based SKIPOGH Study
Source: PLoS One. 2014 Mar 24;9(3):e92522. doi: 10.1371/journal.pone.0092522 (PMC3963885; doi:10.1371/journal.pone.0092522)
Supplement: Table S1 — Mean ambulatory and office systolic blood pressure (SBP), diastolic blood pressure (DBP), and heart rate (HR) by blood pressure category. (DOCX) [file pone.0092522.s001.docx]

**Supplementary Table 1**. Mean ambulatory and office systolic blood pressure (SBP), diastolic blood pressure (DBP), and heart rate (HR) by blood pressure category

|  | Normotension (n=386) | White-coat hypertension (n=17) | Masked hypertension (n=103) | Sustained hypertension (n=146) | P^a^ |
| --- | --- | --- | --- | --- | --- |
| 24-hour SBP (mmHg) | 116.4 (0.4) | 117.5 (2.1)* | 127.2 (1.0) | 124.0 (0.9) | <0.001 |
| Daytime SBP (mmHg) | 120.2 (0.4) | 121.3 (2.3)* | 132.0 (1.0) | 128.3 (1.0) | <0.001 |
| Night-time SBP (mmHg) | 104.4 (0.4) | 105.1 (2.2) | 112.6 (1.1) | 110.7 (1.0) | <0.001 |
| Office SBP (mmHg) | 113.5 (0.5) | 132.9 (2.4)*** | 117.6 (1.0) | 126.4 (1.4)^§§§^ | <0.001 |
| 24-hour DBP (mmHg) | 74.9 (0.2) | 75.0 (1.4) | 83.4 (0.5) | 78.8 (0.7)^§§§^ | <0.001 |
| Daytime DBP (mmHg) | 77.6 (0.2) | 77.6 (1.5) | 87.2 (0.5) | 81.8 (0.7)^§§§^ | <0.001 |
| Night-time DBP (mmHg) | 66.7 (0.3) | 66.8 (1.2) | 71.8 (0.6) | 69.5 (0.6)^§^ | <0.001 |
| Office DBP (mmHg) | 73.5 (0.3) | 82.7 (1.7)*** | 77.7 (0.7) | 79.2 (0.8)^§§^ | <0.001 |
| 24-hour HR (mmHg) | 76.3 (0.4) | 77.0 (1.8) | 80.9 (0.8) | 76.0 (0.7)^§§§^ | <0.001 |
| Daytime HR (mmHg) | 80.3 (0.5) | 80.8 (1.8) | 85.1 (0.9) | 79.8 (0.7)^§§§^ | <0.001 |
| Night-time HR (mmHg) | 64.2 (0.4) | 65.7 (2.2) | 68.1 (0.8) | 64.6 (0.6)^§§^ | <0.001 |
| Office HR (mmHg) | 66.5 (0.5) | 70.2 (1.6) | 69.1 (1.0) | 67.0 (0.7) | 0.018 |

Data are mean (standard error). Analyses are adjusted for age, sex, body mass index, and study centre. ^a^Comparison between the four blood pressure categories, * P < 0.05 vs. normotension, ** P < 0.01 vs. normotension, *** P < 0.001 vs. normotension, ^§^ P < 0.05 vs. masked hypertension, ^§§^ P < 0.01 vs. masked hypertension, ^§§§^ P < 0.001 vs. masked hypertension
